# Outcome measurements in orthopedic

**DOI:** 10.4103/0019-5413.30523

**Published:** 2007

**Authors:** Mohit Bhandari, Brad Petrisor, Emil Schemitsch

**Affiliations:** Departments of Surgery, Divisions of Orthopedic Surgery, McMaster University, Hamilton, Ontario and University of Toronto, Toronto, Ontario, Canada

**Keywords:** Evidence-based medicine, outcomes, research

## Abstract

The choice of outcome measure in orthopedic clinical research studies is paramount. The primary outcome measure for a study has several implications for the design and conduct of the study. These include: 1) sample size determination, 2) internal validity, 3) compliance and 4) cost. A thorough knowledge of outcome measures in orthopedic research is paramount to the conduct of a quality study. The decision to choose a continuous versus dichotomous outcome has important implications for sample size. However, regardless of the type of outcome, investigators should always use the most ‘patient-important’ outcome and limit bias in its determination.

## TYPES OF OUTCOME MEASURES

Investigators have a variety of options when considering outcomes for their studies. Regardless of the specific outcome measure used, outcomes should be “patient-important” and as objective as possible. Mortality is one example of an important and objective outcome measure. However, the majority of orthopedic research focuses upon return to function or measures other than death. Thus, investigators should be familiar with instruments that measure patient function or quality of life. Jackowski and Guyatt^1^ have summarized the key issues in the use of such measures [Table 1]. One of the choices that investigators face when trying to identify an appropriate measure is whether to use generic or disease-specific instruments to measure health status.

**Table 1:** Modes of HRQOL administration

Mode of administration	Advantages	Disadvantages
Interviewer	Maximal response rate Can clarify questions Higher completion rate Control over who is the respondent Control over the order of questions	Costly Interviewer bias Reporting bias Characteristics of the interviewer (voice inflections, age, race, gender) may introduce bias
Telephone	Greater response rate than mail-out Relatively inexpensive Relatively quick data collection Interviewer can probe for incomplete answers Data collector can get clarification for ambiguous answers	Excludes those without access to a telephone Voice inflections of the interviewer may introduce bias
Mail-out	Relatively inexpensive No bias introduced through the interviewer May reach more respondents Respondents can take time to locate certain information	Response rates generally low Possibility of bias due to non-response No control over who is the respondent May misunderstand the question May miss questions (incomplete) Questionnaire may be lost in the mail Excludes illiterate, less educated, handicapped and non-English speaking populations
Self	Maximal response rate Inexpensive	May misunderstand the question May miss questions (incomplete)
Proxy	Can collect information on patients who otherwise are not represented Response may differ from target

A generic instrument is one that measures general health status inclusive of physical symptoms, function and emotional dimensions of health. An example of a generic instrument includes the Short Form-36. A disadvantage of generic instruments however, is that they may not be sensitive enough to be able to detect small but important changes. Disease-specific measures are tailored to inquire about the specific physical, mental and social aspects of health affected by a disease (e.g. arthritis). An example of a disease-specific instrument includes the Western Ontario McMaster Osteoarthritis Index.

The most commonly used generic instrument in the orthopedic surgical literature is the Short Form-36 (SF-36). The SF-36 is a multi-purpose, short-form health survey consisting of 36 questions.^2^,^3^ The SF-36 has proven useful in surveys of general and specific populations, comparing the relative burden of diseases and in differentiating the health benefits produced by a wide range of different treatments.^2^,^3^ The experience to date with the SF-36 has been documented in nearly 4,000 publications; citations for those published in 1988 through 2000 are documented in a bibliography covering the SF-36 and other instruments in the "SF" family of tools.^2^,^3^

The SF-36 contains multi-function item scales to measure eight domains: physical function (10 items); role physical (four items); bodily pain (two items); general health (five items); vitality (four items); social functioning (two items); role emotional (four items); and mental health (five items). The two summary measures of the SF-36 are the physical component summary and the mental component summary. The scores for the multi-function item scales and the summary measures of the SF-36 vary from zero to 100, with 100 being the best possible score and zero being the lowest possible score. The SF-36 takes less than 15min to complete. It can be self-administered or interview-administered. The SF-36 is available in number languages. To use the SF-36, permission must be obtained through Quality Metric (www.SF-36.org).

Utility or performance measures are a unique form of generic instrument that measure health status by quantifying wellness on a continuum anchored by death and optimum health. Assessment of health utility is rooted in decision theory, which models the decision-making process expected of rational individuals when faced with uncertain outcomes. Through placement on a continuum with anchors of death and full health, preference measurement provides a means to compare alternative interventions, patient populations and diseases and is particularly useful when attempting to measure the cost-effectiveness of competing interventions in which the cost of an intervention is related to the number of quality-adjusted life-years (QALYs) gained.

## LIMITING BIAS IN OUTCOMES EVALUATION[Table 2]^2^

**Table 2:** Guidelines for interpreting a study using HRQOL

Has the researcher clearly stated the objectives of the study? Has the role of HRQOL in meeting these objectives been defined? Has the instrument demonstrated validity? Is there a reference made or description of how the instrument was developed? Does the instrument demonstrate face validity? Has the instrument been shown to be valid (content, construct, criterion) in a similar population and disease severity to that of the current study? Can validity and reliability be generalized to the current population and disease? Has the instrument demonstrated reliability? Has the instrument been shown to be reliable over repeated administrations (test re-test) to a stable population, similar in characteristics and disease severity to that of the current study? If more than one rater was involved, was inter-rater reliability established? If a proxy was involved, has the reliability of responses provided by a proxy and the patients been established for this population? Is the instrument sufficiently responsive? Has the instrument demonstrated the ability to detect small but important clinical changes? Are the results of the study valid? Did the author state, a priori, the desired detectable effect size? Has the author provided sufficient evidence or argument for choosing this effect size (clinical importance)? Has the author provided a sufficient description of how the questionnaire was administered? Were data collectors/patients/physicians blinded to the treatment, intervention, exposure or disease being studied? Were patients similar between groups before the intervention? If questionnaires were mailed, is there an adequate comparison of the characteristics of responders and nonresponders? Was the analysis of data appropriate? Were all participants accounted for?

Bias in the measurement of outcomes can be minimized by the use of validated outcome measures, objective outcome measures, blinded assessment of outcomes and independent adjudication of outcomes. Whenever possible, an outcome measure should be blinded. By blinding, the outcome assessor should not be aware of the treatment allocation of the patient in a clinical study. In many surgical trials, however, blinding is impossible and investigators must use alternative methods to minimize bias. In such situations, the outcome measure can be independently adjudicated. By this, we mean that the outcome should be determined an ‘independent’ person or group of individuals who are not otherwise involved in the study. The operating surgeon should not be the individual evaluating outcomes of his or her own patients. When outcomes (e.g. radiographic fracture healing) are subjective in their determination, independent adjudication of one or more persons is an excellent way to limit bias.

## SAMPLE SIZE IMPLICATIONS AND OUTCOME MEASURES

### Outcome measurement and sample size

This section focuses on the choice of an outcome measure and sample size. The statistical power of a study is the probability that it will find a difference between two treatments when one actually exists. By convention, investigators set the acceptable study power to 80% (i.e. 20% chance of false-positive results). Small studies are at risk of being underpowered (study power <80%). Surgeons must endeavor to optimize the study power when they anticipate a small sample size for their studies. The choice of the main outcome variables may play a crucial role in such circumstances.

**Figure 1:**
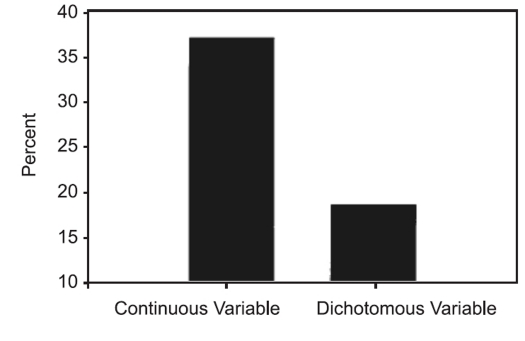
Proportion of adequately powered studies (>80%) with choice of outcome variable

Bhandari *et al* evaluated the impact of the choice of outcome variable on the statistical power in trials of orthopedic trauma.^4^ They hypothesized that small studies with continuous outcome variables (time to fracture union) would achieve higher estimates of study power than those that reported dichotomous outcome variables (% union rates). In a review of 196 RCTs published in 32 medical journals Bhandari *et al* identified a total of 19,942 patients. Study sample sizes ranged from 10 to 662 patients. The vast majority of the studies were conducted at only one center (99.0% or 194/196) and focused upon interventions related to fracture repair (99.0% or 194/196). Fractures of the hip were the primary focus of over one-third of the included studies (34.2% or 67/196). These authors identified 76 studies (39%) with sample sizes of 50 patients or less. Two groups were formed: 29 studies reported continuous outcomes and 47 studies reported dichotomous outcomes. The mean sample size of the studies in each group was similar (*P*>0.05). Those studies that reported continuous outcomes had a significantly greater study power than those studies that reported dichotomous outcomes (*P*=0.042). Twice as many studies that reported continuous outcomes achieved conventionally acceptable study power (80% or more) than those that reported dichotomous outcomes (37% vs. 18.6%, respectively, *P*=0.04) Figure 1.

The power of a statistical test is typically a function of the magnitude of the treatment effect, the designated Type I error rate (α, risk of false-positive result) and the sample size (*n*). When designing a trial, investigators can decide upon the desired study power (typically 80%) and calculate the necessary sample size to achieve this goal. If investigators are conducting a post-hoc power analysis after the completion of the study, they will take the actual sample size used to calculate the study's power.

Moher and colleagues identified 383 randomized trials published in the top medical journals *JAMA, New England Journal of Medicine* and *The Lancet*. Although Moher *et al* did not compare the statistical power and the type of outcome variable, they evaluated 70 trials with negative results and found that 68% lacked acceptable statistical power (80%).^5^ Lochner and colleagues identified 117 randomized trials in orthopedics with a negative result (nonsignificant result) and reported that over 90% lacked sufficient statistical power to make definitive conclusions.^6^ Of the small randomized trials in this review, we identified 78% that were underpowered.

In conclusion, the prevalence of published studies that fail to meet acceptable standards of statistical power is widespread. Surgeons can limit this problem by carefully selecting the outcome variable to optimize the study power and obviate the need for large samples of patients.

Continuous variables are significantly better suited to improving statistical power in small trials than dichotomous variables.

## SAMPLE SIZE CALCULATION

Even at best, a sample size calculation is based upon the best available “guestimate” of treatment difference between treatment groups.

### Comparing two means (continuous variable)^7^–^12^

Let's consider a study that aims to compare pain scores in patients with arthroplasty versus internal fixation in patients with displaced hip fractures. Previous studies using the pain score have reported standard deviations for trauma patients of 12 points. Based upon previous studies, we want to be able to detect a difference of 5 points on this pain score between treatments. Thus, the number of patients required per treatment arm to obtain 80% study power (β=0.20) at a 0.05 alpha level of significance is as follows:

$$n_{1}\;=\;n_{2}\;=2\left(\sigma^{2}\right){\left(z_{1-\alpha /2}\;+\;z_{1-\beta }\right)}^{2}/{∆}^{2}$$

where

n_1_ = sample size of Group one

n_2_ = sample size of Group two

∆ = difference of outcome parameter between groups (5 points)

σ = sample standard deviations^12^

Z_1-α/2_ = z_0.975_ = 1.96 (for α=0.05)

Z_1-β_ = z_0.80_ = 0.84 (for β=0.2)

From the equation above, our proposed study will require 90 patients per treatment arm to have adequate study power n_1_ = n_2_ = 2(12^2^) (1.96 + 0.84)^2^ / 5^2^ = 90.

Reworking the above equation, the study power can be calculated for any given sample size by transforming the above formula and calculating the z-score:

$$z_{1-\beta }\;=\;{\left(n_{1}\left({∆}^{2}\right)/2\left(\sigma^{2}\right)\right)}^{1/2}\;-\;z_{1-\alpha /2}$$

The actual study power that corresponds to the calculated z-score can be looked up in readily available statistical literature^6^ or on the internet (keyword: “z-table”). From the above example the z-score will be 0.84 = (90(5^2^)/2(12^2^))^1/2^ - 1.96 for a sample size of 90 patients. The corresponding study power for a z-score of 0.84 is 80%.

### Comparing binomial proportions (percentages for dichotomous variables)

Let's now assume that we wish to change our outcome measure to differences in secondary surgical procedures between operatively and nonoperatively treated ankle fractures. We consider a clinically important difference to be 5%. Based upon the previous literature, we estimate that the secondary surgical rates in operatively and nonoperatively treated ankles will be 5% and 10%, respectively. The number of patients required for our study can now be calculated as follows:

$$n_{1}\;=\;n_{2}\;=\;{\left[{\left(2p_{m}q_{m}\right)}^{1/2}z_{1-\alpha /2}\;+\;{\left(p_{1}q_{1}\;+\;p_{2}q_{2}\right)}^{1/2}\;z_{1-\beta }\right]}^{2}/{∆}^{2}$$

where

n_1_ = sample size of Group one

n_2_ = sample size of Group two

p_1_, p_2_ = sample probabilities (5% and 10%)

q_1_, q_2_ = 1 - p_1_, 1 - p_2_ (95% and 90%)

p_m_ = (p_1_ + p_2_)/2 (7.5%)

q_m_ = 1 - p_m_ (92.5%)

∆ = difference = p_2_ - p_1_ (5%)

Z_1-α/2_ = z_0.975_ = 1.96 (for α=0.05)

Z_1-β_ = z_0.80_ = 0.84 (for β=0.2)

Thus, we need 433 patients per treatment arm to have adequate study power for our proposed trial.

n_1_ = n_2_ = [(2 × 0.075 × 0.925)^1/2^ × 1.96 + (0.05 × 0.95 + 0.1 × 0.9)^1/2^ × 0.84^2^] / 0.05^2^= 433

Reworking the above equation, the study power can be calculated for any given sample size by transforming the above formula and calculating the z-score:

$$z_{1-\beta }\;=\;{\left(n\;\left({∆}^{2}\right)\right)}^{1/2}\;-\;{\left(2p_{m}q_{m}\right)}^{1/2}z_{1-\alpha /2})/{\left(p_{1}q_{1}\;+\;p_{2}q_{2}\right)}^{1/2}$$

From the above example the z-core will be 0.84 = ((433 × 0.05^2^)^1/2^ - (2 × 0.075 × 0.925)^1/2^ × 1.96) / (0.05 × 0.95 + 0.1 × 0.9)^1/2^ for a sample size of 433 patients. The corresponding study power for a z-score of 0.84 is 80%.

### Using confidence intervals for sample size calculation

It can also be useful to calculate the precision of a study based on the above sample size calculation. Precision is defined as the width of the 95% confidence interval (CI). Being 95% confident means that if we repeat the study an unlimited number of times, the true difference between groups will be included in the CI in 95% of the samples. For any power and clinically relevant or hypothesized difference (Δ) the predicted confidence interval can be calculated using this formula:

$$\mathrm{Predicted}\;95\%\;\mathrm{CI}\;=\;\mathrm{observed}\;\mathrm{differrence}\;\pm \;0.7\;{∆}_{0.80}$$

$$\mathrm{Predicted}\;\mathrm{Precision}\;=\;2*0.7{∆}_{0.80}\;=\;1.4{∆}_{0.80}$$

where

∆_0.80_= true difference for which there is 80% power.

Often, choosing an expected difference between two groups can be arbitrary. An alternative method to determine an expected difference can be derived from using 95% confidence intervals. For example, rather then hypothesizing a 5% difference between operative and nonoperative treatment of ankle fractures we might be more comfortable stating that we will not accept a confidence interval for an observed difference that is wider than 7%. Thus we can work backwards from our predicted confidence interval to calculate the expected difference between groups:

$$0.07\;=\;1.4{∆}_{0.80}$$

$${∆}_{0.80}\;=\;0.07/1.4\;=\;0.05$$

Now we can use the sample size calculation for the proportions above to calculate the number of patients required for our study.

Calculating the precision illustrates the trade-off between the magnitude of the hypothesized or clinically relevant difference used in the sample size calculation and the likelihood of finding a statistically significant difference. Choosing a higher hypothesized difference decreases the required number of studied subjects, but it also increases the predicted 95% confidence interval, which then is more likely to include 0 and therefore yielding statistically not significant results. While it is tempting to “hypothesize” a larger difference of the primary outcome parameter in order to decrease the required sample size, it is therefore advisable to choose a realistic difference when calculating the required sample size. Also, the benefit of calculating the predicted precision is that it may be easier to understand for a nonstatistician that the primary outcome parameter would be within a specific range (in this example 7%) rather than dealing with the more abstract concept of study power.

Depending on the magnitude of the required study subjects, the investigators have to evaluate the feasibility of single versus multi-center study and the enrollment period. Finally, investigators should not confuse clinical significance with statistical significance. Any result will be statistically significant if enough study subjects are used.

## CONCLUSION

A thorough knowledge of outcome measures in orthopedic research is paramount to the conduct of a quality study. The decision to choose a continuous versus dichotomous outcome has important implications for sample size. However, regardless of the type of outcome, investigators should always use the most ‘patient-important’ outcome and limit bias in its determination.
